# P-1291. High Prevalence of Carbapenemase genes within Class 1 Integrons among Carbapenem-Resistant Enterobacterales in Southern Taiwan

**DOI:** 10.1093/ofid/ofaf695.1479

**Published:** 2026-01-11

**Authors:** Susan Shin-Jung Lee, Hui-Ling Hsia, Yi-Ting Lee, Hsi-Hsun Lin

**Affiliations:** Kaohsiung Veterans General Hospital, Kaohsiung, Kaohsiung, Taiwan (Republic of China); Kaohsiung Veterans General Hospital, Kaohsiung, Kaohsiung, Taiwan (Republic of China); Kaohsiung Veterans General Hospital, Kaohsiung, Kaohsiung, Taiwan (Republic of China); Kaohsiung Veterans General Hospital, Kaohsiung, Kaohsiung, Taiwan (Republic of China)

## Abstract

**Background:**

Class 1 integrons are frequently associated with antimicrobial resistance genes in clinical bacterial isolates. However, the prevalence of carbapenemase genes within class 1 integrons among carbapenem-resistant *Enterobacterales* (CRE) remains underexplored. This study aimed to investigate the prevalence of carbapenemase genes and antimicrobial resistance gene cassettes within class 1 integrons among CRE isolates in Southern Taiwan.

**Table 1.**
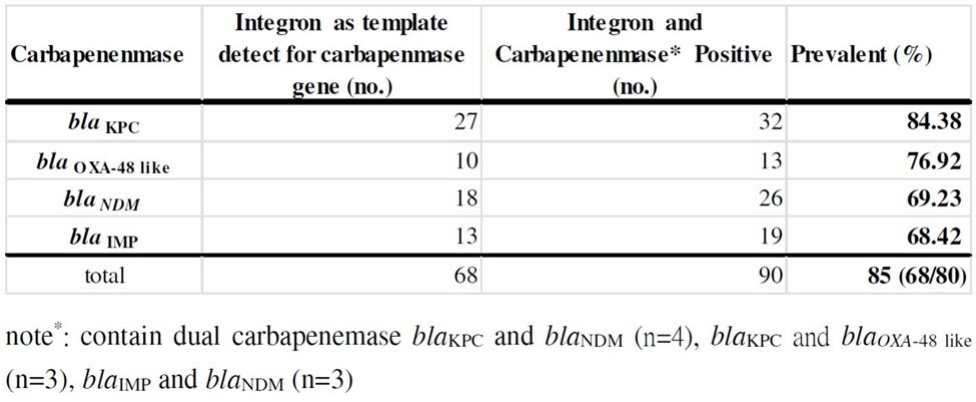
Prevalence of Integron cassettes carrying carbapenemase (CP) genes in CRE in southern Taiwan. (N=80)

**Figure 1.**
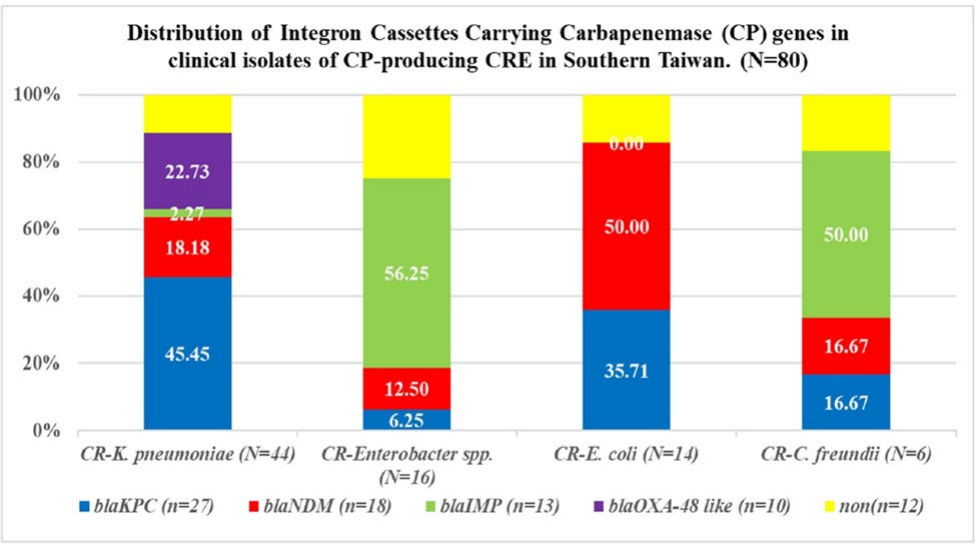
Distribution of Class 1 Integron Cassettes Carrying Carbapenemase (CP) genes in clinical isolates of CP-producing CRE in Southern Taiwan (N=80)

**Methods:**

A total of 101 CRE clinical isolates were collected between 2020 and 2023. DNA was extracted, and PCR was performed to detect class 1 integrons, which then served as templates for carbapenemase gene detection and sequence analysis. Sequencing was conducted using an Applied Biosystems 3730xl automatic sequencer or Nanopore amplicon NGS, with subsequent alignment against GenBank databases.

**Figure 2.**
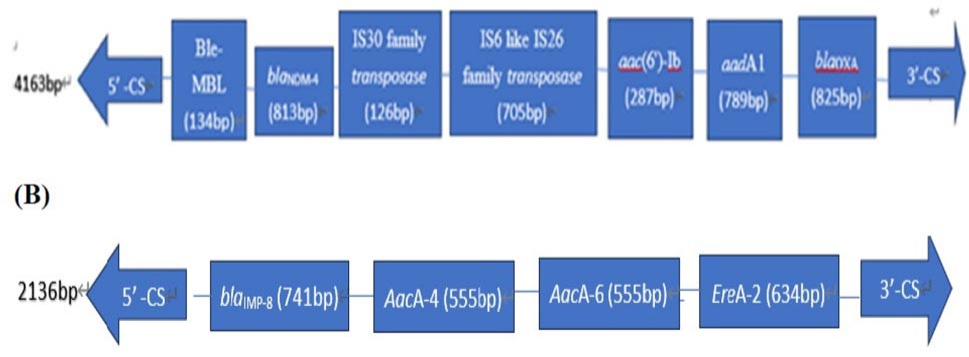
The size and structure of resistance gene cassettes between two conserved ends (5′CS and 3′CS) of the class 1 integrons detected in the clinical isolates of Enterobacteriales. (A) no.CS297-CP-CR-K. pneumoniae (B) no.CS299-CP-CR- E. hormaechei

**Results:**

In our previous study, a high prevalence of integrons were detected among CP-CRE isolates (79.2%, 80/101). Further analysis using PCR revealed that 85% (68/80) of these integron-positive strains harbored antibiotic resistance gene cassettes associated with carbapenemases. Among these, *bla*_KPC_ was the most prevalent (84.4%, 27/32), followed by *bla*_OXA-48-like_ (76.9%, 10/13), *bla*_NDM_ (69.2%, 18/26), and *bla*_IMP_ (68.4%, 13/19). The highest expression of integron gene cassettes associated with carbapenemase genes by species included *bla*_KPC_ in CR-*K. pneumoniae* (45.5%), *blaIMP* in CR-*Enterobacter spp*. (56.3%), *bla*_NDM_ in CR-*E. coli* (50%), and *bla*_IMP_ in CR-*C. freundii* (50%). Nanopore amplicon NGS sequencing identified integron gene cassettes Ble-MBL-1-*bla*_NDM-4_-IS30 family transposase-IS6-like element IS26 family transposase-*aac*(6')-Ib-*aad*A1-783- *bla*_OXA_ in CS297-CP-CR-*K. pneumoniae* and another *bla*_IMP-8_-*Aac*A-4-*Aac*A6- *ere*A2 in CS299-CP-CR- *E. hormaechei*.

**Conclusion:**

This study demonstrates a high prevalence of class 1 integron gene cassettes carrying carbapenemase genes among CP-producing CRE. Nanopore amplicon next-generation sequencing (NGS) confirmed the co-localization of *blaNDM-4* and *blaIMP-8* with integron gene cassettes in CP-CR-*K. pneumoniae* and CP-CR-*E. hormaechei*, respectively, supporting their role in enhanced multidrug resistance dissemination.

**Disclosures:**

All Authors: No reported disclosures

